# *OAZ-t*/*OAZ3* Is Essential for Rigid Connection of Sperm Tails to Heads in Mouse

**DOI:** 10.1371/journal.pgen.1000712

**Published:** 2009-11-06

**Authors:** Keizo Tokuhiro, Ayako Isotani, Sadaki Yokota, Yoshihisa Yano, Shigeru Oshio, Mika Hirose, Morimasa Wada, Kyoko Fujita, Yukiko Ogawa, Masaru Okabe, Yoshitake Nishimune, Hiromitsu Tanaka

**Affiliations:** 1 TANAKA Project, Center for Advanced Science and Innovation, Osaka University, Suita, Osaka, Japan; 2 Animal Resource Center for Infectious Diseases, Research Institute for Microbial Diseases, Osaka University, Suita, Osaka, Japan; 3 Laboratory of Functional Anatomy, Faculty of Pharmaceutical Sciences, Nagasaki International University, Sasebo, Nagasaki, Japan; 4 Department of Bioscience and Biotechnology, Faculty of Bioenvironmental Science, Kyoto Gakuen University, Kameoka, Kyoto, Japan; 5 Department of Hygiene Chemistry, Ohu University School of Pharmaceutical Sciences, Koriyama, Fukushima, Japan; 6 Molecular Biology, Faculty of Pharmaceutical Sciences, Nagasaki International University, Sasebo, Nagasaki, Japan; 7 Microbiology, Faculty of Pharmaceutical Sciences, Nagasaki International University, Sasebo, Nagasaki, Japan; 8 Research Collaboration Center on Emerging and Re-emerging Infections, Research Institute for Microbial Diseases, Osaka University, Suita, Osaka, Japan; The University of North Carolina at Chapel Hill, United States of America

## Abstract

Polyamines are known to play important roles in the proliferation and differentiation of many types of cells. Although considerable amounts of polyamines are synthesized and stored in the testes, their roles remain unknown. Ornithine decarboxylase antizymes (OAZs) control the intracellular concentration of polyamines in a feedback manner. OAZ1 and OAZ2 are expressed ubiquitously, whereas OAZ-t/OAZ3 is expressed specifically in germline cells during spermiogenesis. OAZ-t reportedly binds to ornithine decarboxylase (ODC) and inactivates ODC activity. In a prior study, polyamines were capable of inducing a frameshift at the frameshift sequence of OAZ-t mRNA, resulting in the translation of OAZ-t. To investigate the physiological role of OAZ-t, we generated OAZ-t–disrupted mutant mice. Homozygous OAZ-t mutant males were infertile, although the polyamine concentrations of epididymides and testes were normal in these mice, and females were fertile. Sperm were successfully recovered from the epididymides of the mutant mice, but the heads and tails of the sperm cells were easily separated in culture medium during incubation. Results indicated that OAZ-t is essential for the formation of a rigid junction between the head and tail during spermatogenesis. The detached tails and heads were alive, and most of the headless tails showed straight forward movement. Although the tailless sperm failed to acrosome-react, the heads were capable of fertilizing eggs via intracytoplasmic sperm injection. OAZ-t likely plays a key role in haploid germ cell differentiation via the local concentration of polyamines.

## Author Summary

Polyamines are essential for cell proliferation and differentiation, but their role in these processes is unknown. Ornithine decarboxylase antizymes (OAZs) are enzymes that control the concentration of polyamines in cells. To elucidate the role of one of these enzymes, OAZ-t, in the regulation of polyamine concentration during sperm formation, we generated mutant mice in which the OAZ-t gene was disrupted. When we observed sperm from the mice lacking a functional Oaz-t gene, we found that the sperm heads separated easily from the tails, indicating that OAZ-t is essential for the formation of a rigid junction between the head and tail during sperm development. Many of the headless tails could continue swimming, but they were unable to participate in the signaling processes required for successful fertilization. However, tailless heads could produce healthy pups when injected into unfertilized eggs. Such a phenotype has not been previously found. The mutant mice evoked rare cases of infertile human patients whose sperm behaves in a proper fashion. Our study underscores the importance of research into the processes of spermatogenesis and fertilization.

## Introduction

As many as 15% of human couples [1] are infertile, and male infertility is associated with about half of these cases. A decrease in sperm production has recently been reported [1]. Although advances in medical technology have allowed some infertile couples to have children, more than half of all infertility is idiopathic [1]. Because unresolved environmental problems such as global pollution might be causing endocrine disruption, a thorough understanding of the basic mechanisms of germ cell differentiation is critical for development of infertility treatments. To elucidate the molecular mechanisms of spermiogenesis, we isolated many cDNA clones specifically expressed in haploid germ cells using a subtracted haploid germ cell-specific cDNA library [2]. One of them (TISP15) encoded the Ornithine decarboxylase antizyme (OAZ) known to control the intracellular concentration of polyamines [3],[4]. Full-length TISP15, also known as OAZ in testis (OAZ-t/OAZ3), was specifically expressed in haploid germ cells [4],[5]. Polyamines, such as putrescine, spermidine, and spermine, are essential for cell proliferation and differentiation via binding to nucleic acids as cations [6],[7]. The actual function of polyamines is not entirely clear although significant amounts of polyamines are synthesized and stored in the testes [3],[8]. The biosynthesis of polyamines is regulated strictly by many proteins via the key enzyme of ornithine decarboxylase (ODC). OAZ is a major regulator of ODC [9]. Upon stimulation with polyamines, OAZ protein is translated by programmed +1 frameshifting to inhibit ODC activity specifically, and the OAZ–ODC complex drives the rapid degradation of ODC by the 26S proteasome [10]–[15]. OAZ belongs to a conserved gene family with at least three members in the vertebrate lineage. OAZ1 and OAZ2 are expressed ubiquitously in all somatic tissues [9],[16],[17]. In male germ cell, the RNA expression of somatic OAZ1 was decreased during the later stages of haploid germ cell differentiation [4]. Further analysis of OAZ-t revealed that polyamines are capable of inducing a frameshift at the frameshift sequence in OAZ-t mRNA [4], resulting in the translation of OAZ-t, as is the case for somatic OAZ1 [10]. The transfection of OAZ-t cDNA inhibits ODC activity in HEK293 cells [11]. OAZ-t may play important roles in the regulation of polyamine concentration in spermiogensis. To clarify the roles of OAZ-t specifically expressed in haploid germ cells, we produced the OAZ-t-disrupted mice and analyzed the effect of the disappearance of OAZ-t.

## Results

### OAZ-t/OAZ3 homozygous mutant males are infertile

**Figure 1:**
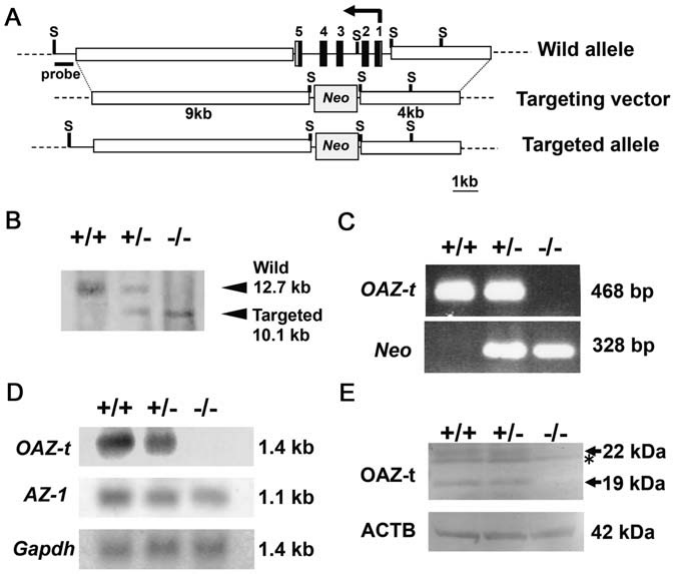
Generation of *OAZ-t* knockout mice. (A) Schematic representation of the methods used for gene targeting of the *OAZ-t* genome. The gene targeting construct contains *Neo* (open box) between the 4-kb 5′-arm and 9-kb 3′-arm (thick lines). As a result, exons 1–5 were replaced with *Neo*. Exon 1 includes the first methionine. Arrows indicate the transcriptional direction of *OAZ-t*. *S* indicates *Sac*I restriction sites. (B) The targeted allele was identified by the Southern blotting of genomic DNA digested with *Sac*I using a probe created from the 3′ fragment. (C) The mice were genotyped by PCR using two sets of primers: one set for the amplification of the *Neo* gene (*Neo*) and one set for the amplification of the *OAZ-t* gene. +/+: wild-type; +/−: heterozygous mutant; −/−: homozygous mutant. (D) Analysis of gene expression by northern blotting. No *OAZ-t* transcripts were detected in the testes of *OAZ-t* homozygous mutant mice. The same membrane was rehybridized with *AZ-1* or *Gapdh* cDNA as a control. (E) Western blotting of testicular lysates from adult mice using anti-OAZ-t polyclonal antibodies. OAZ-t was not detected in the lysates from the homozygous mutant mice (22 kDa). The 19-kDa band may be a degradation product of OAZ-t. The asterisk indicates a non-specific band. β-Actin (ACTB) was used as a control.

A targeting vector was constructed (Figure 1A) and homologous recombination was used to generate embryonic stem (ES) cell clones that were heterozygous for the *OAZ-t* mutation. To produce chimeric mice, transgenic ES cells were injected into blastocysts that were subsequently implanted into pseudopregnant mice. Correct recombination was confirmed by Southern blotting (Figure 1B) and PCR (Figure 1C). No *OAZ-t* expression was detected in the testes of the homozygous null *OAZ-t* mutant mice by northern (Figure 1D) or western blotting (Figure 1E). Crossing of heterozygous mutant pairs produced the expected numbers of wild-type, heterozygous, and homozygous offspring, according to classical Mendelian inheritance patterns. Matings between homozygous *OAZ-t* knockout males and wild-type females did not result in any successful pregnancies over a period of more than three months of continuous cohabitation, although vaginal plugs were observed in the paired wild-type females (Table 1). All heterozygous *OAZ-t* males and homozygous females were fertile (Table 1). Neither the homozygous null mutant nor the heterozygous males exhibited a significant differentiation in body mass (Table S1). Female body mass and the weights of various organs, including the testes and seminal vesicles in the adult *OAZ-t* homozygous mutant mice, were identical to those in the heterozygous mice (Table S1). The serum testosterone levels and polyamine contents in the adult *OAZ-t* homozygous mutant male mice were identical to those in the wild-type mice (Table S1 and Table S2).

**Table 1:** Fertility rates among the mutant mice.

Genotype		+/−	−/−
Male	Fertility (no. of fertile males/no. of males)	10/10	0/10
	Litter size (avg. no. of newborn pups)	6.9±1.8	0
Female	Fertility (no. of fertile females/no.of females)	10/10	10/10
	Litter size (avg. no. of newborn pups)	6.4±0.3	6.5±0.5

Values are means±SEM.

### OAZ-t is essential in sperm formation

**Figure 2:**
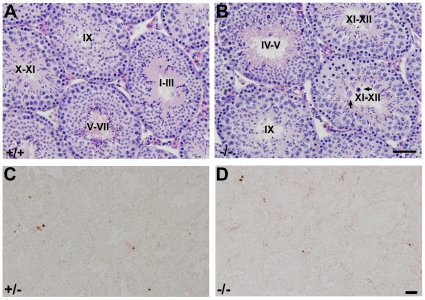
Histological analysis and TUNEL staining of testicular sections of mutant testes. Hematoxylin- and eosin-stained cross-sections of heterozygous (A) or homozygous mutant (B) testes from adult mice are shown. Greek numerals indicate the stages of seminiferous tubules, as defined by Russell et al. [18]. Morphologically normal spermatogenesis rarely occurred in the mutant testes. Arrowheads indicate a few germ cells located irregularly at the luminal region of the mutant seminiferous tubules. TUNEL staining of cross-sections of heterozygous (C) or homozygous mutant (D) testes from adult mice are shown. Apoptotic signals were rarely observed in the heterozygous and homozygous mutant testes. Bar = 50 µm.

**Figure 3:**
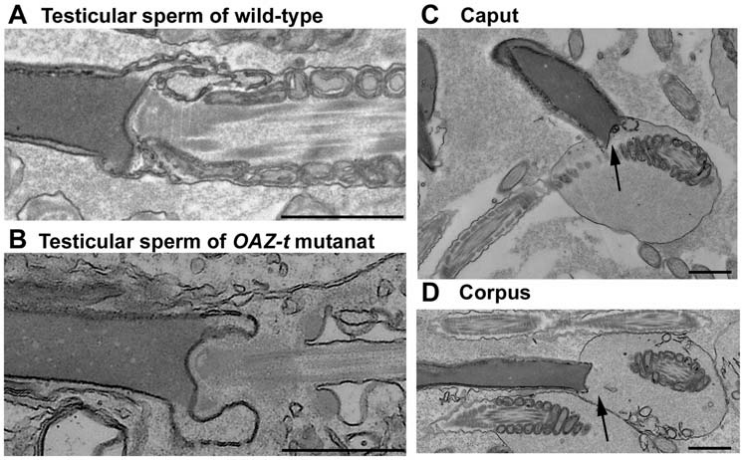
Electron microscopic observation of morphology for sperm maturation in testis and epididymis. The head-tail junction in testicular sperm of wild-type (A) and OAZ-t null mutant (B) is shown. The sperm tail in the homozygous *OAZ-t* mutant epididymis (C,D) was improperly arranged at the head tail junction. Arrows indicate the abnormal head-tail jounction. Bar = 1.0 µm (A–D).

**Figure 4:**
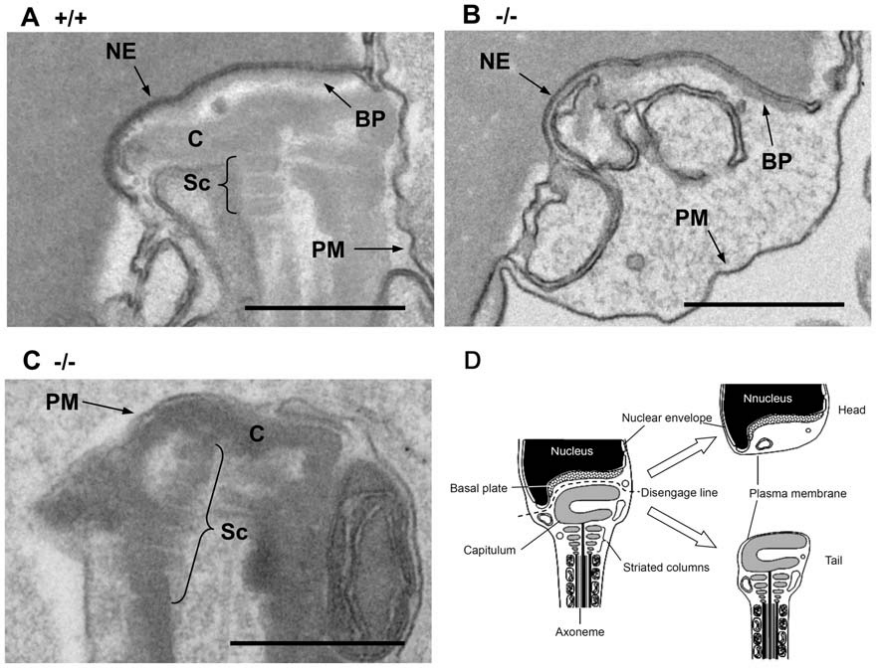
Morphology of mature sperm in the cauda epididymis. The image in (A) shows the neck region of a sperm cell from a wild-type mouse. In the implantation fossa, several typical components of the connection between head and tail can be identified: the basal plate (BP), the capitulum (C), and striated columns (Sc). NE: nuclear envelope, PM: plasma membrane. The image in (B) shows the neck region of a separated head obtained from a mutant mouse. The basal plate (BP) is apparent at the outer nuclear membrane, but the remaining connecting components (*i.e.*, the capitulum and striated columns) are absent. The image in (C) shows the distal end of the separated tail. Note that the capitulum (C) and striated columns (Sc) are present. Bar = 0.5 µm. (D) Schematic presentation of mature sperm in OAZ-t null mutant.

Histological analyses of the testes by light microscopy showed normal morphology (Figure 2A and 2B). In mice, the spermatogenic cycle that occurs in each tubule of the seminiferous epithelium is divided into 12 stages, and the germ cells in the seminiferous tubules are enclosed by Sertoli cells [18]. Spermatogonia, spermatocytes, and spermatids were systematically arranged in the seminiferous tubules of the heterozygous mutant and wild-type testis: spermatogonia were found in the tubule walls, whereas spermatids were located in the tubule centers and spermatocytes were observed between the two (Figure 2A). Tubules with an abnormal arrangement of cells undergoing spermatogenesis were rarely observed in the homozygous mutant mice (Figure 2B). To identify apoptotic cells, we performed terminal deoxynucleotidyltransferase-mediated dUTP nick end-labeling (TUNEL) staining using an *in situ* apoptosis detection kit (Takara, Shiga, Japan) according to the manufacturer's instructions. There was no statistical difference in signal between testicular sections prepared from the homozygous and heterozygous mutant mice (Figure 2D and 2E). Fully differentiated sperm were observed in the seminiferous tubules by light microscopy and there was no difference in weight between the homozygous and heterozygous mutant testes (Table S2). Electron microscopic analysis revealed that flagellar formation and nuclear condensation occurred normally in spermatids until step nine (data not shown) and in elongated spermatids in the testes of homozygous mutants (Figure 3A and 3B). However, the direction and location of each flagellum was arranged incorrectly at the caudal pole of the nucleus during maturation in the epididymis (Figure 3C and 3D, and Figure S1). The mitochondria and the outer dense fibers were arranged normally, with few mitochondria to drop out in the cytoplasm. Separation of the sperm head and tail was observed in spermatozoa in the cauda epididymis (Figure 4). In wild-type sperm, the components connecting the sperm head to the flagellum were observed as described in earlier studies [19]–[22]. The basal plate attaching to the outer membrane of the nuclear envelope was identified in wild-type and mutant sperm (Figure 4A and 4B). The capitulum, consisting of electron-dense material, was observed between the basal plate and striated columns, which continued to the axoneme (Figure 4A). In the mutant mouse, the capitulum and striated columns were not apparent in the cytoplasm of the separated head (Figure 4B), but they were observed in the separated tail (Figure 4C). These results strongly suggest that separation occurred between the basal plate and capitulum. Disengagement of the tail from the head was accompanied by plasma membrane, and both stumps were sealed (Figure 4B–4D).

### Heads and tails of sperm were active

**Figure 5:**
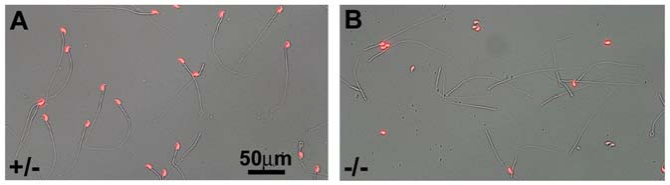
Characteristics of *OAZ-t*-null sperm. Epididymal sperm were cultured in TYH medium for 1 h. The *OAZ-t*-null heads were easily separated and aggregated. The nuclei were stained with PI after PFA fixation. Analysis of *OAZ-t*-null sperm by optical microscopy.

Although similar numbers of sperm were recovered from the cauda epididymides of the homozygous and heterozygous mutants, almost all of the sperm heads from the homozygous null mice were detached from tails during incubation in culture medium (Figure 5). Meanwhile, the headless tails showed surprisingly normal energetic swimming ability (Videos S1 and S2). They maintained their swimming ability even after 15 h of incubation. The movement of the separated tails looks normal, although no sign of hyperactivation is evident. We also examined the viability of the tailless sperm heads by staining with propidium iodide (PI). The heads could be considered as maintaining membrane integrity because they were resistant to PI staining (Table 2). It is well known that sperm have no fertilizing ability upon ejaculation, undergoing physiological (capacitation) and morphological change (acrosome reaction) before acquiring the ability to fuse with eggs [23]. Acrosome reaction was known to be artificially induced by a treatment of sperm with calcium ionophore A23187. Therefore, we examined whether or not the tailless heads which were found to be “alive” could respond to the ionophore and undergo induced acrosome reaction. As shown in Table 2, these tailless sperm heads showed no response to the ionophore and acrosome reaction did not take place. The role of OAZ-t in acrosome reaction is not clear at present. However, if we recall research indicating the existence of acrosome reaction-related molecules such as AKAPs [24],[25] and CatSpers [26] in tails, it is possible to assume that the induction of acrosome reaction in the head requires signals from tails [27].

**Table 2:** Acrosome reaction.

Genotype	1 hour	3 hours	6 hours	Calcium Ionophore*	Living sperm**(%)
+/+	39.7±3.2	48.8±2.1	63.0±13.7	95.3±2.5	60.66±0.35
+/−	30.0±5.3	39.7±2.1	50.0±7.5	81.3±3.8	60.87±0.67
−/−	11.3±0.6	28.3±2.5	33.7±5.7	34.3±3.8	61.55±0.95

Values are means±SD; n = 3 assays.

*: Calcium ionophore A23187 was added to the medium after 3 hours.

**: Propidium iodide-negative sperm was measured by flow cytometer after 6 hours.

### Sperm of OAZ-t mutant mice can produce viable embryos

The homozygous mutant sperm were not able to fertilize eggs by in vitro fertilization (IVF) assays (data not shown). Therefore, we injected sperm heads derived from heterozygous and homozygous *OAZ-t* mutant mice into cytoplasm of unfertilized eggs (ICSI). Twenty-two and fifteen two-cell-stage embryos were obtained from the heterozygous and homozygous *OAZ-t* mutant sperm, respectively. They were transferred to the oviducts of pseudopregnant females and three healthy pups were sired by each genotype. Thus the infertile nature of OAZ-t null sperm is not derived from the defects in the quality of the head itself.

## Discussion

Previous study showed that the polyamine concentration in the germ cells increased after meiotic division, whereas the level of ODC activity declined [28],[29]. It has been proposed that Sertoli cells provide polyamines to germ cells [30]. OAZ-t plays to regulation of polyamine concentration in spermiogenesis instead of OAZ1 and 2. OAZ1 binds to ODC with about a three-fold higher potency than OAZ2 [31]. OAZ1 accelerates proteasomal ODC degradation, whereas OAZ2 does not [31]. OAZ1, OAZ2, and OAZ3/OAZ-t indeed differ in their effect on ODC activity in vitro or in bacteria [4],[16],[32],[33]. The concentrations of polyamines in testis and sperm were not affected by the disruption of *OAZ-t*. Since the activity of ODC was not regulated only by OAZ-t but by other regulatory proteins such as OAZ inhibitor (AZI) [33], it was assumed that the polyamines amount was kept normal by other factors. OAZ-t was dispensable in regulation of total cellular polyamine concentration. A previous study showed that exogenous primary amines induced head-tail dissociation as a result of the separation of the inner and outer nuclear envelope membranes adjacent to the tail basal plates [21]. These results indicated that the concentration of primary amines affected construction at the head−tail junction of sperm. Because polyamines are alkanes and include primary amines, the segregation of sperm heads and tails may be caused by a change in the local concentration of polyamines.

The orthologue of the *OAZ-t* gene is reported in human. One team investigated the relationship between OAZ-t polymorphism and male infertility. The researchers found one Pro164Ser mutation in one of the azoospermic patients but it is not clear if this substitution affects OAZ-t function. The researchers did not claim an association of OAZ-t polymorphism to human male infertility [34]. In previous studies, decaudated tails and decapitated heads lacking the implantation fossa and basal plate at the caudal pole of the nucleus were observed in infertile patients [35],[36]. This phenotype is consistent with the null mutation of OAZ-t in mice. OAZ-t may thus be one of the genes responsible for decaudated tails and decapitated heads in human sperm, although the neckpieces of human and mouse sperm are not identical. Our *OAZ-t*-disrupted mouse line may offer insight into the mechanism of spermatogenesis.

## Materials and Methods

### Ethics statement

All animal experiments conformed to the Guide for the Care and Use of Laboratory Animals and were approved by the Institutional Committee of Laboratory Animal Experimentation (Nagasaki International University, Nagasaki, and the Research Institute for Microbial Diseases, Osaka). The mice were kept under controlled temperature and lighting conditions throughout the experiments and were provided with food and water *ad libitum*.

### Construction of the targeting vector and the production of *OAZ-t* targeting mice

The *OAZ-t* targeting construct was created by the amplification of a homologous 4.0-kb 5′-arm and 9.0-kb 3′-arm using 129Sv genomic DNA as the template. The primers used to amplify the arms were designed to incorporate synthetic enzyme sites at both ends. The amplified fragments were digested to create sticky ends and the clone was sequentially ligated into the poly-linker cloning sites on either side of the neomycin resistance gene in the targeting vector backbone. The targeting vector contained the neomycin resistance gene and a thymidine kinase gene, both under the control of the PGK promoter. The vector plasmid was linearized by *Not*I digestion prior to electroporation into W9.5 ES cells. Of 720 G418 gancyclovir-resistant clones screened, two were found to have undergone homologous recombination correctly by Southern blot analysis. The four targeted cell lines were injected into C57BL/6J blastocysts, resulting in the birth of male chimeric mice. Highly chimeric males were mated with C57BL/6J wild-type females to generate F1 offspring, half of which were heterozygous for the targeted allele. Of the two ES cell lines injected, both lines produced a high percentage of chimeras that entered the germline. Heterozygous F1 males were then crossed to C57BL/6 females to obtain heterozygous F2 animals. The heterozygous F2 animals were bred to produce homozygous mutants and to check for Mendelian inheritance.

### Breeding of the mice

The mice were bred and maintained in our laboratory animal facilities and used in accordance with the guidelines for the care and use of laboratory animals set forth by the Japanese Association for Laboratory Animal Science. Genomic DNA was extracted from the tails of the mice using standard procedures. Southern blotting was conducted to determine the site of integration for the gene trap sequence in the *oaz-t* locus and to genotype the mice. A 3′ external probe was generated by PCR (primers 5′-CATGATGTCACTGACTCTTTCC-3′ and 5′-CAATGGAAGATGGAAGAATATG-3′) from mouse genomic DNA. Genomic DNA samples (10 µg) were digested with *Sac*I and electrophoresed on 0.8% agarose gels. All hybridizations were performed using standard protocols. The mice were genotyped by PCR using two sets of primers (Figure 1C) as follows: one set of primers (5′-ATCTGGACGAAGAGCATCAGGGG-3′ and 5′-CCTCAGAAGAACTCGTCAAGAAG-3′) to amplify the *Neo* gene and one set of primers (5′-TCAGGCCTTGGATCAAGGCAACCG-3′ and 5′- CATACTCCAGTGTTGCTGTCAAGC -3′) for the *oaz-t* gene. To examine the expression of *oaz-t*, northern blotting was performed according to the manufacturer's instructions using PerfectHyb (Toyobo, Osaka, Japan) [4]. Western blotting was performed according to a previously-described protocol [4].

### Morphological observation of the testes and epididymal sperm

For morphological observation, testes were fixed in Bouin's solution, embedded in paraffin, and sectioned at a thickness of 8 µm. Deparaffinized sections were stained with hematoxylin and eosin. Sperm from the cauda epididymis were cultured in TYH medium (119 mM NaCl, 4.8 mM KCl, 1.7 mM CaCl_2_, 1.2 mM KH_2_PO_4_, 1.0 mM MgSO_4_, 25 mM NaHCO_3_, 5.6 mM glucose, 0.5 mM sodium pyruvate, and 4 mg/ml BSA) for 30 min, spotted onto glass slides, and dried.

### Electron microscopy

Testes and the caput, corpus, and cauda epididymis of wild-type and mutant mice were fixed in fixative consisting of 4% paraformaldehyde, 2% glutaraldehyde, 0.05 M HEPES-KOH buffer (pH 7.4) and 0.02% CaCl_2_ for 2 h at room temperature. Spermatozoa from the cauda epididymis were suspended in PBS and centrifuged at 1000× *g* for 5 min. The pellets were fixed with the same fixative as mentioned above. All fixed samples were post-fixed with 1% reduced osmium for 1 h, dehydrated in a series of graded ethanol solutions, and embedded in Epon. Thin sections were stained with lead citrate and examined with a Hitachi H7650 electron microscope.

### Analysis of the sperm

The status of the acrosome was evaluated by staining with FITC–PNA (Sigma-Aldrich), which binds the outer acrosomal membrane. Sperm samples were dried on glass slides and fixed with 70% methanol at −20°C for 5 min after incubation in TYH medium at 37°C in a humidified incubator containing 5% CO_2_/95% air. A23187 (Sigma-Aldrich) was added at a final concentration of 10 µM to induce the acrosome reaction [37]. Fluorescence-activated cell sorting (FACS) analysis was used to monitor the activity of the sperm following PI staining. ICSI was performed as described [38]. Briefly, sperm collected from the epididymides of the mice were suspended in 12% polyvinylpyrrolidone (360 kDa; PVP) and decapitated with a Piezo pulse (Prime Tech Ltd., Tokyo, Japan). The detached heads were then introduced into the cytoplasm of unfertilized cumulus-free eggs. After being incubated in kSOM for 24 h [39], the eggs were transplanted at the two-cell stage to pseudopregnant females.

### Statistical analysis

Differences between the experimental and control conditions were compared using one-way analysis of variance with Fisher's protected least significant difference test. Significant differences (*P*<0.01) are discussed here.

## Supporting Information

Figure S1 Electron microscopic observation of the morphology of wild-type epididymal sperm. Bar = 1 µm.
(1.36 MB TIF)

Table S1 Body weight, organs' weights, and serum testosterone levels of the male mice.
(0.03 MB DOC)

Table S2 Polyamine contents in testis and epididymis.
(0.04 MB DOC)

Video S1 Motility of the OAZ-t +/− sperm in TYH medium. Sperm were collected from the cauda epididymides, placed in chamber slides, and observed in real time by light microscopy after 5 h. The OAZ-t +/− sperm exhibited normal motility.
(0.17 MB MOV)

Video S2 Motility of the OAZ-t null sperm in TYH medium. The OAZ-t null sperm were separated easily into heads and flagella, and the headless sperm swam mightily.
(0.23 MB MOV)
