# Supplementary figures and images for: OAZ-t/OAZ3 Is Essential for Rigid Connection of Sperm Tails to Heads in Mouse
Source: PLoS Genet. 2009 Nov 6;5(11):e1000712. doi: 10.1371/journal.pgen.1000712 (PMC2763286; doi:10.1371/journal.pgen.1000712)

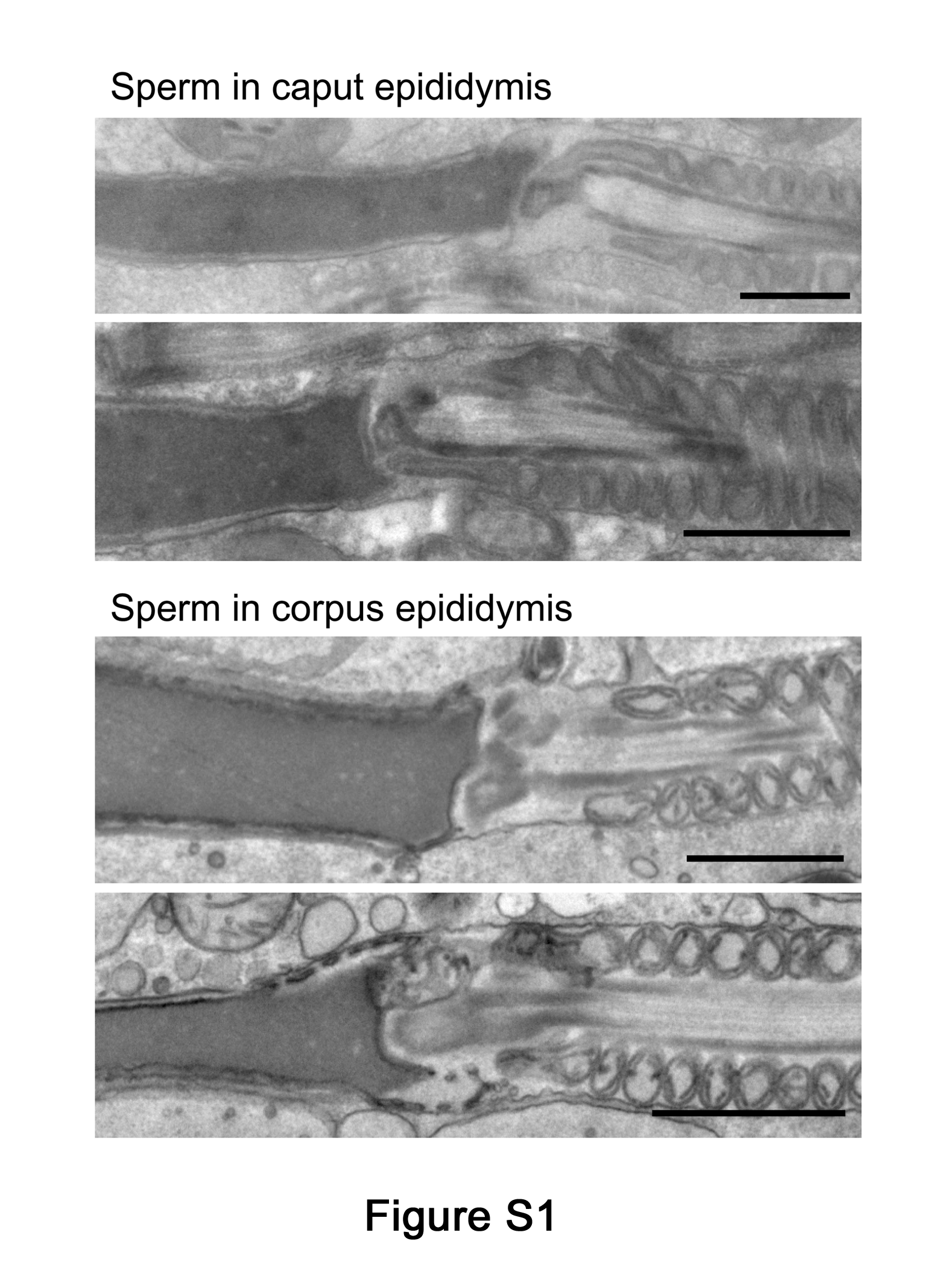

Supplement: Figure S1 — Electron microscopic observation of the morphology of wild-type epididymal sperm. Bar = 1 µm. (1.36 MB TIF) [file pgen.1000712.s001.tif]
