# Supplementary material for: OAZ-t/OAZ3 Is Essential for Rigid Connection of Sperm Tails to Heads in Mouse
Source: PLoS Genet. 2009 Nov 6;5(11):e1000712. doi: 10.1371/journal.pgen.1000712 (PMC2763286; doi:10.1371/journal.pgen.1000712)
Supplement: Table S1 — Body weight, organs' weights, and serum testosterone levels of the male mice. (0.03 MB DOC) [file pgen.1000712.s002.doc]

| **Table S1**  **Body weight, organs weights, and serum testosterone levels of the male mice** | | | |
| --- | --- | --- | --- |
| Parameter | Value for *OAZ-t* ^+/-^mice | Value for *OAZ-t* ^-/-^ mice |  |
| Body weight |  |  |  |
| male | 28.0±0.7 | 24.2±1.2 |  |
| female | 25.0±0.8 | 22.8±0.9 |  |
| Wet weight of organs/ Body weight |  |  |  |
| Testis | 3.32±0.14 | 3.69±0.09 |  |
| Epididymis | 1.61±0.06 | 1.60±0.06 |  |
| Seminal vesicle | 15.87±3.81 | 11.38±0.92 |  |
|  |  |  |  |
| Testosterone level (ng/ml) | 3.17±1.25 | 3.47±1.35 |  |

All values are means ± SEM. N=6 mice per genotype.

Significant differences (*P*, .01) are discussed here.
