# Supplementary material for: OAZ-t/OAZ3 Is Essential for Rigid Connection of Sperm Tails to Heads in Mouse
Source: PLoS Genet. 2009 Nov 6;5(11):e1000712. doi: 10.1371/journal.pgen.1000712 (PMC2763286; doi:10.1371/journal.pgen.1000712)
Supplement: Table S2 — Polyamine contents in testis and epididymis. (0.04 MB DOC) [file pgen.1000712.s003.doc]

**Table S2 Polyamine content**  （nmol/mg protein）

|  | Genotype | *N*^1^-AcSPD* | Putrescine | Spermidine | Spermine |
| --- | --- | --- | --- | --- | --- |
|  | +/+ | 0.17±0.04 | 0.58±0.04 | 1.13±0.30 | 0.51±0.23 |
| Epididymis | +/- | 0.24±0.06 | 0.56±0.03 | 1.65±0.34 | 0.68±0.27 |
|  | -/- | 0.22±0.06 | 0.64±0.16 | 1.49±0.44 | 0.40±0.20 |
|  | +/+ | 0.47±0.06 | 0.26±0.05 | 1.65±0.08 | 0.83±0.09 |
| Testis | +/- | 0.56±0.13 | 0.38±0.02 | 1.78±0.08 | 0.70±0.05 |
|  | -/- | 0.73±0.04 | 0.40±0.18 | 1.80±0.34 | 0.72±0.06 |

The tissues were washed with ice-cold PBS and disrupted by ultrasonication in 0.4 N perchloric acid. After centrifugation at 18,000 x *g* for 20 min, the supernatant was stored at -20 ˚C until the polyamine assay. The pellet was used to measure the protein content. The polyamine concentration in the acid extract was determined by high-performance liquid chromatography (Shimadzu LC-6A; Shimadzu, Kyoto, Japan) using a fluorescence detector. The polyamines were separated on an STR ODS-II column (4.6 x 150 mm, particle size 5 μm; Shimadzu Techno-Research, Kyoto, Japan; Matsui-Yuasa *et al. Biol. Interact.* **81**, 233–242 [1992]). The polyamine contents are expressed as nanomoles per milligram of protein. Protein precipitated with 0.4 N perchloric acid was solubilized in 0.2 M NaOH. After centrifugation at 18,000 x *g* for 20 min, the protein content of the supernatant was measured by the bicinchoninic acid assay (Smith, P.K. *et al*. *Anal Biochem.* **150**, 76–85 [1985]) using a Pierce BCA Protein Assay Kit (Thermo Fisher Scientific Inc., Rockford, IL) with bovine serum albumin as a standard.

The values indicate the means±SD; n = 3 assays.

Significant differences (*P* < 0.01) are discussed here.

**N*^1^-acetylspermidine
